# Real‐time gestational‐age estimation at 11–13 weeks using artificial intelligence on non‐targeted ultrasound sweeps

**DOI:** 10.1002/uog.70335

**Published:** 2026-09-14

**Authors:** G. Eisenkolb, D. Wright, A. Syngelaki, G. Soraci, S. Medrano Záciga, K. H. Nicolaides

**Affiliations:** ^1^ Fetal Medicine Research Institute, King's College Hospital London UK; ^2^ Institute of Health Research University of Exeter Exeter UK; ^3^ Department of Women & Children's Health School of Life Course & Population Sciences, Faculty of Life Sciences & Medicine, King's College London London UK

**Keywords:** Accuracy, crown–rump length, deep learning, fetal medicine, first trimester, gestational age, ultrasound

## Abstract

**Objective:**

To assess the accuracy of gestational‐age estimation using an artificial intelligence (AI) system applied to non‐targeted ultrasound sweeps at 11 + 0 to 13 + 6 weeks' gestation.

**Methods:**

This was an observational study of singleton pregnancies undergoing routine first‐trimester ultrasound in July 2025 to August 2025. Gestational age was estimated using crown–rump length (CRL), measured independently by two experienced sonographers blinded to the other's results, and by an AI system applied twice per examination. Comparisons were made using mean absolute error (MAE) and Bland–Altman analysis. A hierarchical measurement error model was used to account for uncertainty in CRL‐based estimates and to estimate accuracy relative to a latent true gestational age, with Bayesian inference.

**Results:**

Overall, 285 women with a singleton pregnancy were included. The AI model estimated gestational age in all cases, with a median acquisition time of 23 s for the first AI measurement. The MAE between AI‐ and CRL‐based gestational age was 2.1 (95% CI, 1.9–2.3) days for single measurements and 2.0 days when averaging both AI estimates. Limits of agreement were wider for AI‐based estimates than for CRL‐based estimates, indicating lower precision. When accounting for measurement error in CRL, the estimated MAE for AI was lower (1.8 days for single and 1.6 days for duplicate measurements). CRL‐based estimates showed lower variability than AI‐based estimates. Within‐operator variation was the main source of error in AI measurements.

**Conclusions:**

AI‐based gestational‐age estimation performs well in the first trimester but is less precise than gestational‐age estimation using CRL measured by experienced sonographers. Measurement error models give a clearer picture of performance by accounting for variability in CRL and show that the accuracy of AI‐based estimates is better than suggested by simple comparisons. © 2026 The Author(s). *Ultrasound in Obstetrics & Gynecology* published by John Wiley & Sons Ltd on behalf of International Society of Ultrasound in Obstetrics and Gynecology.

## INTRODUCTION

Accurate determination of gestational age (GA) is critical for all aspects of obstetric care. While GA was traditionally estimated from the first day of the last menstrual period (LMP), it is now most commonly determined using first‐trimester ultrasound measurement of the fetal crown–rump length (CRL)^1^. Precise assessment of CRL is important for both aneuploidy screening and early‐pregnancy risk assessment for pre‐eclampsia^2^, ^3^, and also for classifying fetuses as small‐, appropriate‐ or large‐for‐gestational age later in pregnancy^4^, ^5^.

First‐trimester CRL measured until 13 + 6 weeks is recommended by USA and UK guidelines as the most accurate method for dating a pregnancy^6^, ^7^. The World Health Organization (WHO) recommends that at least one ultrasound scan is performed before 24 weeks' gestation^8^. In developed healthcare settings such as the UK, pregnancy dating in the first trimester represents standard care, with approximately 85% of women undergoing ultrasound assessment between 11 + 0 and 13 + 6 weeks' gestation^9^. However, in many low‐ and middle‐income countries (LMICs), GA estimation is limited by access to trained personnel and suitable devices. Even in well‐resourced settings, obtaining accurate CRL measurements requires considerable operator skill and time.

Artificial intelligence (AI) models have demonstrated calculation of GA estimates comparable to those of conventional biometry, including in cases in which ultrasound images were acquired by novice operators^10^, ^11^. The performance of AI models is typically summarized using simple metrics such as mean absolute error (MAE) relative to a reference standard. Measurement error models provide a more informative approach by explicitly accounting for different sources of variation and avoiding the need to assume a definitive ground truth. In study designs with replicated measurements across multiple operators, variability can be decomposed into within‐ and between‐operator components, allowing assessment of both repeatability and generalizability across operators. This makes it possible to identify the main sources of disagreement between methods^12^. When implemented within a Bayesian framework, these models also allow uncertainty in GA estimates to be quantified.

In this study, we assessed the approach of Benson *et al*.^13^, which applies a deep learning system to non‐targeted ultrasound sweeps, focusing on pregnancy dating between 11 + 0 and 13 + 6 weeks. We evaluated performance of the AI system using both conventional MAE calculations and a measurement error model, and compared the results, accounting for CRL measurement error.

## METHODS

### Study population and design

This was an observational study of women with a singleton pregnancy who presented for a routine 11–13‐week scan at King's College Hospital, London, UK, in July 2025 to August 2025. All women attending for a routine first‐trimester visit during the study period were offered the opportunity to participate in the trial, with an aim of recruiting 300 participants. Recruitment was closed after obtaining 307 women. Pregnancies conceived via *in‐vitro* fertilization (IVF) were excluded from the analysis. Diagnosis of a major fetal abnormality also led to exclusion from the study. At the 11–13‐week visit, we recorded maternal characteristics, including age, height, weight, body mass index (BMI), racial origin (White, Black, South Asian, East Asian or mixed) and medical history. We performed an ultrasound scan for determination of GA using the fetal CRL^1^ and for diagnosis of major fetal abnormalities^14^. The study was approved by the National Health Service Health Research Authority (reference: 25/LO/0426) and all study participants provided written informed consent for inclusion.

### Ultrasound examination and use of AI system

All ultrasound examinations were performed according to standardized protocols by sonographers with Fetal Medicine Foundation (FMF) certification for performance of the first‐trimester scan. Ultrasound examinations were conducted using a GE Voluson S10 or E22 system (GE Healthcare, Zipf, Austria), either transabdominally (3–7.5‐MHz curvilinear transducer) or transvaginally (3–9‐MHz transducer) when adequate views could not be obtained transabdominally. Fetal CRL was measured independently by two sonographers who were blinded to the other's results, and GA was determined using the CRL measurement obtained by the first sonographer.

The AI system (GE Healthcare) was implemented on a tablet device connected to the ultrasound system to receive a live video feed during transabdominal scanning. The deep‐learning model used for GA estimation has been described previously^13^. The AI system was used twice per examination, once at the beginning and once at the end, to generate two independent GA estimates. During AI image acquisition, undirected scanning of the fetus was performed until a result was produced. To account for the intended use of the AI system by novice ultrasound operators, the scan consisted of real‐time ultrasound sweeps intended to capture fetal position, fetal heartbeat and a broad range of views rather than predefined standard planes, with no image freezing or view optimization performed. The tablet device displayed a progress ring showing the percentage of progress and AI image acquisition was stopped once the progress ring showed 100%. GA (in weeks + days) and time to obtain a GA estimate were recorded. Sonographers were blinded to AI outputs, and no standard CRL measurements were obtained during AI video acquisition.

Patients were scanned by a total of 45 sonographers, of whom 32 also performed the AI assessments. For each patient, two independent CRL measurements and two AI‐based estimates were obtained, enabling the application of a measurement error modeling framework. For AI‐based GA, the same operator performed two measurements, whereas for CRL‐based GA, two different operators each performed one measurement.

### Statistical analysis

First, for comparison with previous studies, GA estimated by the AI system was compared with that derived from standard CRL measurement using MAE and the cumulative distribution of the absolute error (AE), with the mean of the two CRL‐based estimates taken as the reference standard. Uncertainty was summarized using confidence intervals. Variability in both AI‐based and CRL‐based GA estimates was assessed using Bland–Altman analyses of paired measurements, performed separately for AI‐ and CRL‐based estimates. These analyses represent within‐operator variability for the AI method and between‐operator variability for the CRL method.

Second, to account for uncertainty in CRL‐based GA estimates, a hierarchical measurement error model was analyzed within a Bayesian framework, with the true GA (τ) treated as an unobserved latent variable. GA estimated from the CRL was modeled as an imperfect measurement of τ, with random effects to account for within‐ and between‐operator variability. AI‐based GA estimates were modeled analogously as functions of τ, incorporating corresponding within‐ and between‐operator random effects. Intercept and slope terms were included to allow for calibration differences between AI‐ and CRL‐based estimates, and a subject‐specific random effect was included for AI measurements to allow for differences between AI and conventional ultrasound that were not explained by the systematic calibration relationship between the two methods. This allows the agreement between AI‐ and CRL‐based estimates to vary between subjects.

MAEs relative to the latent τ estimated from the measurement error model were compared with MAEs obtained using CRL as the reference standard, as well as with previously published results^13^, to assess the impact of accounting for measurement error in CRL‐based GA.

To characterize measurement variability in clinically interpretable terms, we specified a hierarchical measurement error model relating the unobserved τ to observed measurements.

To ensure model identifiability, CRL was taken as the reference measurement, such that the expected CRL‐based estimate equals τ, although CRL itself was still modeled as subject to measurement error via between‐ and within‐operator variability. In contrast, AI‐based GA estimates were allowed to deviate systematically from τ through a calibration term, as well as through random error. The variability of this distribution arises from three additive sources of error: (i) subject–method interaction (random effect), variability associated with applying the measurement method to a specific individual; (ii) between‐operator variability, systematic differences between operators, affecting all measurements made by a given operator; and (iii) within‐operator variability, random variation arising from repeated measurements made by the same operator.

For both CRL‐ and AI‐based measurements, variability was decomposed into between‐operator and within‐operator components, reflecting differences between operators and repeatability within operators. In addition, the AI‐based method included a method‐specific error component, representing variability intrinsic to the algorithm when applied to individual cases. This framework separates variability arising from how measurements are performed from variability inherent to the measurement approach.

Statistical analysis was performed in R software (R Foundation for Statistical Computing, Vienna, Austria), and the Bayesian analysis was implemented in WinBUGS using non‐informative priors^15^. Details are provided in Appendix S1.

## RESULTS

During the study period, we initially recruited 307 singleton pregnancies with a live fetus and CRL of 45–84 mm, in which an ultrasound examination was performed between 11 + 0 and 13 + 6 weeks. In total, 285 women that conceived spontaneously were included after exclusion of 22 women that conceived via IVF, resulting in 570 measurements of CRL and 570 results produced by AI. Median maternal age was 33.9 (interquartile range (IQR), 31.0–36.0) years, median maternal BMI was 24.3 (IQR, 22.4–27.6) kg/m^2^, and the racial origin was White in 218 (76.5%) women, Black in 31 (10.9%), South Asian in 19 (6.7%), East Asian in nine (3.2%) and mixed in eight (2.8%). At the time of the ultrasound scan, the median CRL was 63.3 (IQR, 59.3–67.9) mm when obtained by the first sonographer and 62.9 (IQR, 59.5–67.6) mm when obtained by the second sonographer. The median GA estimated by the first CRL measurement was 12.7 (IQR, 12.4–13.0) weeks.

AI‐based estimation of GA successfully returned a result in all cases, with a median time to obtain a GA estimate of 23 (IQR, 18–30) s for the first AI measurement and 19 (IQR, 14–28) s for the second AI measurement.

### Mean absolute error

The MAE between AI‐based GA and CRL‐based GA was 2.1 (95% CI, 1.9–2.3) days for the first AI measurement and 2.2 (95% CI, 2.0–2.3) days for the second AI measurement. Using the average of both AI measurements resulted in a slightly lower MAE of 2.0 (95% CI, 1.8–2.1) days. Figure 1 shows the cumulative AE distribution for AI‐based *vs* CRL‐based GA estimation. In 90% of cases, the AE between AI‐based and CRL‐based GA estimates was within 4.0 days when using the average of both AI measurements. Comparison of the two AI estimates resulted in a MAE of 1.4 days between the first and second AI measurement, with an intraclass correlation coefficient of 0.88 (95% CI, 0.85–0.90), indicating high reproducibility of AI results. Table S1 shows subgroup analyses according to maternal BMI and ethnicity. Although these analyses were limited by small subgroup sizes, no significant differences in model performance based on maternal BMI or ethnicity were observed.

**Figure 1:**
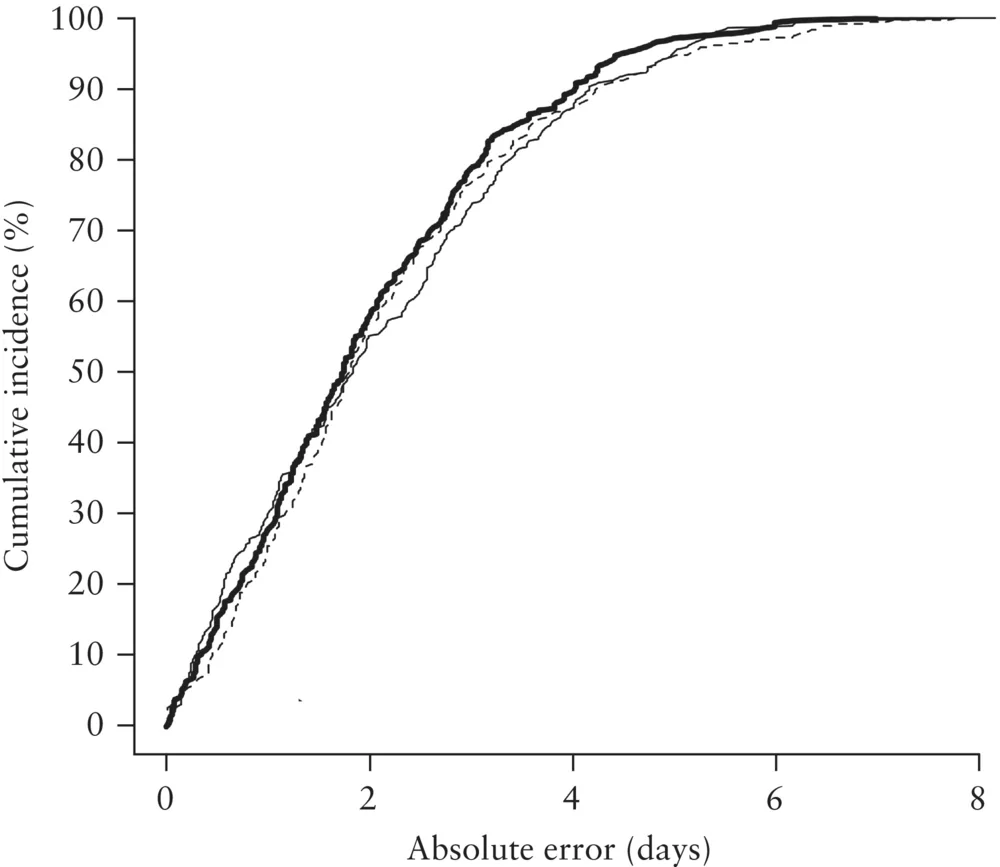
Cumulative incidence of absolute error (in days) between artificial intelligence (AI)‐based gestational‐age estimates and estimates based on the average of two crown–rump length (CRL) measurements. 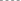, first AI measurement; 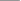, second AI measurement; 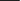, average of first and second AI measurements.

### Bland–Altman analysis

Bland–Altman analyses were performed separately for CRL‐based and AI‐based GA estimates, capturing between‐operator variability for CRL and within‐operator variability for AI. Figure 2 shows scatter plots and Bland–Altman plots of paired measurements, presented separately for GA estimated using CRL and using the AI method.

**Figure 2:**
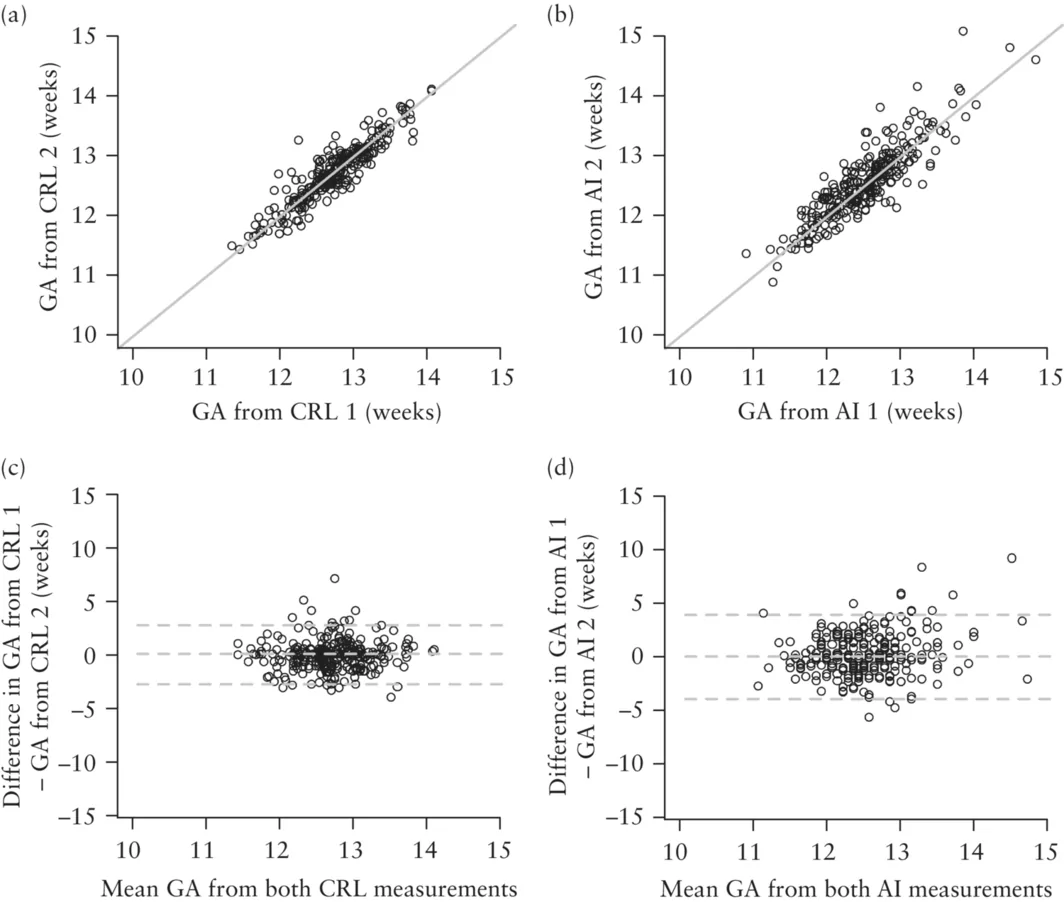
(a,b) Scatter plots comparing gestational‐age (GA) estimates from first (1) and second (2) repeat measurements and (c,d) Bland– Altman plots comparing the difference between GA estimates and their mean, for crown–rump length (CRL) (a,c) and artificial intelligence (AI) (b,d) measurements. Horizontal dashed lines indicate limits of agreement.

The limits of agreement were wider for AI‐based estimates than for CRL‐based estimates, indicating lower precision, as reflected by the greater spread (Figure 2). This is notable as wider limits would ordinarily be expected for between‐operator rather than within‐operator comparisons; here, the within‐method variability of the AI approach exceeds the between‐operator variability of CRL measurements.

### Measurement error modeling

We developed a hierarchical measurement error model relating the latent true GA (τ) to observed measurements, as illustrated in Figure 3. Table 1 shows that CRL measurements had relatively low total variability, largely driven by within‐operator variation, whereas AI‐based estimates had substantially greater total variability, reflecting higher within‐operator variability, an additional method effect component and evidence of systematic bias. Using our measurement error model resulted in MAEs of 1.8 days (single measurement) and 1.6 days (duplicate measurements) when including the AI‐specific method effect. Although the systematic bias of AI relative to CRL contributes to conventional measures of AI performance, it can be addressed through recalibration by subtracting the estimated intercept. The MAEs presented in Table 1 were therefore calculated from the estimated error SDs, both including and excluding the method effect, with the latter assuming that calibration biases can be removed.

**Figure 3:**
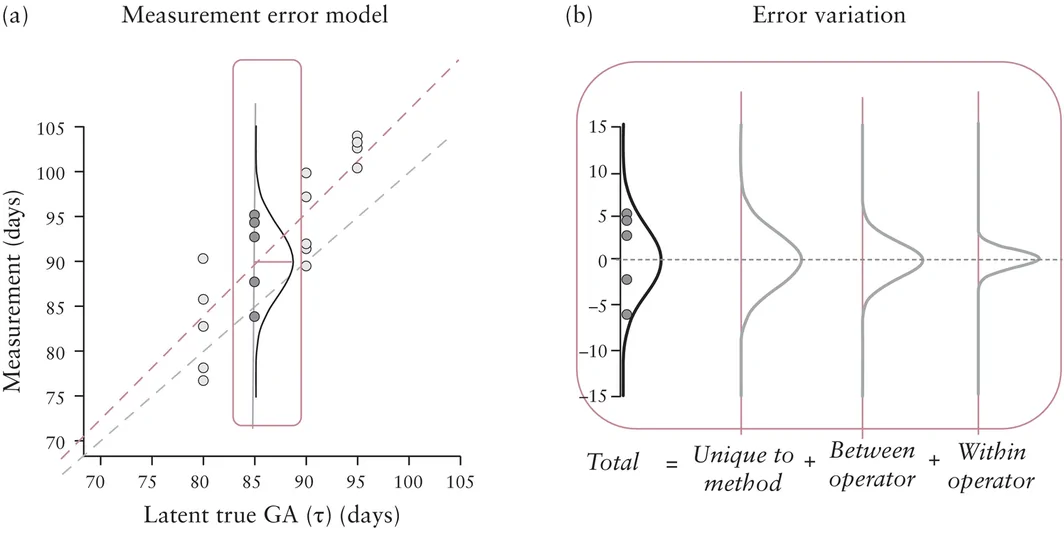
(a) Schematic representation of measurement error model relating latent true gestational age (GA) (τ) to observed measurements. Observed measurements are distributed around mean observed measurement according to a Gaussian (normal) distribution (b). Together, these components define a hierarchical error structure, with the total measurement error corresponding to the convolution (sum) of these independent Gaussian sources. 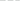, line of identity (perfect agreement between true and observed values); 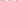, expected (mean) observed measurement for a given value of τ, allowing for systematic bias.

**Table 1:** Posterior summaries from measurement error model for crown–rump length (CRL)‐based and artificial intelligence (AI)‐based gestational‐age (GA) estimates

Parameter	Mean (days)	SD (days)	Median (95% CrI) (days)
Latent true GA (τ)			
Mean	89.01	0.20	89.01 (88.62–89.41)
SD	3.289	0.144	3.284 (3.020–3.585)
CRL‐based estimate			
Between operator SD	0.211	0.097	0.209 (0.042–0.412)
Within operator SD	0.955	0.042	0.954 (0.876–1.042)
Total error SD	0.983	0.042	0.981 (0.905–1.071)
MAE (single)	0.784	0.034	0.783 (0.722–0.855)
MAE (duplicate)	0.570	0.029	0.567 (0.519–0.635)
AI‐based estimate			
Calibration intercept	−1.30	0.14	−1.30 (−1.58 to −1.02)
Method SD	1.645	0.114	1.644 (1.427–1.873)
Between operator SD	0.241	0.172	0.206 (0.030–0.634)
Within operator SD	1.432	0.060	1.430 (1.320–1.557)
Total error SD			
Including method effect	2.204	0.085	2.201 (2.044–2.379)
Excluding method effect	1.462	0.070	1.457 (1.336–1.612)
MAE (single)			
Including method effect	1.759	0.068	1.756 (1.631–1.898)
Excluding method effect	1.167	0.056	1.163 (1.066–1.286)
MAE (duplicate)			
Including method effect	1.561	0.075	1.558 (1.421–1.714)
Excluding method effect	0.840	0.052	0.834 (0.758–0.965)

CrI, credible interval; MAE, mean absolute error.

The method effect component has a different interpretation. Under the original parameterization of the model, it reflects differences between AI‐ and CRL‐based estimates of GA that are not explained by calibration differences or measurement error, with the CRL‐based GA, τ, treated as the reference. However, an alternative interpretation is to treat AI and CRL symmetrically and assume distinct true GAs for the two methods, calculated as $\tau_{\mathrm{AI}}=\tau_{\mathrm{CRL}}+\alpha +\varepsilon$ or equivalently $\tau_{\mathrm{CRL}}=\tau_{\mathrm{AI}}-\alpha -\varepsilon$. In this interpretation, α represents the mean disagreement between true scores and ε represents a random disagreement effect. Thus, there may be intrinsic differences between AI‐based and CRL‐based estimates of GA beyond calibration differences or measurement error. In this framework, the comparison of measurement precision is based on the variability around the respective true scores. For CRL, the SD of the total measurement error was estimated to be 0.983 days, compared with 1.462 days for AI‐based estimates (excluding the method effect). These differences are reflected in the wider limits of agreement observed. Under this assumption, the MAE for the single AI measurement was 1.2 days, and as low as 0.8 days for duplicate AI measurements (Table 1).

### Comparisons

Table 2 shows our results in the context of previously published findings. MAE estimates from our dataset were broadly comparable to those reported by Benson *et al*.^13^, falling within the range observed across different imaging types. Notably, MAE estimates derived using the measurement error model were consistently lower than those obtained when treating CRL‐based estimates as the true GA. This reflects the impact of accounting for measurement error in CRL‐based estimates, which otherwise inflates apparent discrepancies between AI‐based estimates and the reference standard. Averaging duplicate AI measurements results in a modest but consistent reduction in MAE, both under conventional analysis and within the measurement error framework, with the greatest improvement seen in model‐based estimates.

**Table 2:** Mean absolute error (MAE) of gestational age (in days) from previously published findings^13^ and our dataset using both conventional analysis (treating crown–rump length as the ground truth) and a measurement error model

Source	MAE (days)
Benson *et al*.^13^	
Biometry benchmark	2.8
AI static image	1.3
AI videoclips	2.5
Present study	
Conventional analysis	
Single AI measurement	2.1
Mean of both AI measurements	2.0
Measurement error model	
Single AI measurement	
Including method effect	1.8 (1.7–1.9)
Excluding method effect	1.2 (1.1–1.3)
Mean of both AI measurements	
Including method effect	1.6 (1.5–1.7)
Excluding method effect	0.8 (0.8–1.0)

95% credible interval is given in parentheses for measurement error model‐based estimates. AI, artificial intelligence.

## DISCUSSION

### Main findings

This observational study of singleton pregnancies at 11 + 0 to 13 + 6 weeks' gestation has demonstrated that the MAE of AI‐based GA estimation was 2.0 days when compared to CRL‐based assessment and 1.6 days when compared to our measurement error model. The AI system reliably obtained a GA estimate in a median time of 23 s.

However, in our study, pregnancy dating based on the CRL was more accurate than AI‐based estimation of GA. When compared to a statistically derived latent true GA (τ) using the measurement error model, the MAE was < 1 day for the CRL‐based assessment and < 2 days for AI‐based GA estimation. The measurement model revealed that the between‐operator variability for CRL‐based dating was lower than the within‐operator variability for the AI model, highlighting method‐specific inaccuracies of the AI system.

The interpretation of the measurement error model depends on the role assigned to CRL. If CRL is regarded as the reference standard for GA assessment, then the latent true GA is naturally anchored to CRL and any residual disagreement between AI‐based and CRL‐based estimates, beyond calibration differences, is attributed to the AI model. Under this interpretation, AI exhibits both greater measurement variability and an additional method effect relative to CRL.

However, there is also a case for symmetric interpretation. Neither CRL nor AI directly measure GA; rather, both provide estimates based on different sources of information. In this view, disagreement in the method effect between the two methods need not be interpreted as error attributable solely to AI, but may instead reflect genuine differences between the underlying true scores represented by the two approaches. Under this interpretation, the method effect reflects intrinsic differences between AI‐based and CRL‐based assessments of GA rather than a deficiency of either method.

Although these alternative interpretations affect the attribution of disagreement between methods, they do not alter the comparison of measurement precision. The total measurement error SD remained lower for CRL (0.983 days) than for AI (1.462 days) (excluding the method effect), indicating that AI‐based estimates exhibit greater variability around their respective true scores.

### Comparison with the literature

AI models have been developed to date pregnancies during each of the trimesters^10^, ^11^, ^13^, ^16^, ^17^, ^18^. Whereas some systems use still images or videoclips acquired during routine ultrasound examination^18^, many models are trained to work on blind ultrasound sweeps. These are designed to ensure systematic coverage of the maternal abdomen or uterus according to predefined protocols and can be performed by novice ultrasound operators.

A key challenge in evaluating AI performance in first‐trimester pregnancy dating is the absence of a true GA. Some studies use pregnancies conceived via IVF, in which the true GA is known; however, evidence regarding first‐trimester fetal growth in IVF pregnancies is inconsistent, particularly with respect to conception method and embryo transfer type, limiting their suitability as a reference standard^19^, ^20^, ^21^, ^22^. Conversely, treating CRL‐based estimates as the true GA introduces circularity, as it is one of the methods under evaluation.

Several studies focus on second‐ and third‐trimester pregnancy dating, in which both AI‐ and biometry‐based assessments are compared to an observed true GA (either GA defined by first‐trimester CRL or by conception date for IVF pregnancies). Viswanathan *et al*. reported a MAE of 3.1 days for second‐trimester and 4.4 days for third‐trimester AI‐based estimation of GA, which outperformed pregnancy dating according to fetal biometry based on INTERGROWTH‐21st charts^17^. In a more recent study of Stringer *et al*., the MAE for dating using an AI model was 3.2 days compared with 3.0 days when using conventional biometry (INTERGROWTH‐21st or Hadlock) between 14 + 0 and 27 + 6 weeks^11^. However, second‐ and third‐trimester pregnancy dating using conventional ultrasound and fetal biometry is inherently inaccurate as it is based on growth charts with an increasing spread around the median with advancing gestation^23^. Pokaprakarn *et al*. used a blind sweep protocol from 9 weeks onwards in Zambia and the USA, and reported MAEs of 2.1 *vs* 2.0 days for first‐trimester dating using AI *vs* CRL, respectively^10^.

Benson *et al*.^13^ trained the AI model used in our study on over 2 million images obtained from routine ultrasound examinations of 78 531 pregnancies across the UK, Australia and India. For first‐trimester dating between 10 and 14 weeks, they reported a MAE of 1.3 days when the AI model was used on static images. However, testing the same AI model on 36 videoclips resulted in a MAE of 2.5 days. Using the same AI system, we report a MAE of 2.0 days. After accounting for measurement error in CRL, instead of treating CRL‐based estimates as the latent true GA, the MAE was 1.6 days (Table 2). We can only speculate on the reasons for the difference between the reported MAEs for videoclip and still image data. Benson *et al*.^13^ reported that their training dataset consisted of still images obtained during routine clinical scanning. Usually, in a routine clinical examination, only images meeting distinct quality criteria, including appropriate standard planes with optimized magnification, gain, depth and focus, are saved. In contrast, real‐time videos are more likely to contain a wider range of image quality and fetal orientations. To the best of our knowledge, this study is the first to validate the AI system on real‐time ultrasound videos, with results close to the reported MAEs for still images obtained in less than 30 s.

### Strengths and limitations

The primary strength of this study lies in its design, including 285 pregnancies and 45 trained sonographers performing CRL measurements, of whom 32 also used AI‐based methods, generating 570 measurements for both CRL and AI. This supports generalizability across both ultrasound practitioners and AI operators, and enables assessment of within‐ and between‐operator variability.

From a methodological perspective, measurement error models offer important advantages over simple comparisons based on MAE^12^. By accounting for multiple sources of variability, they allow more meaningful comparisons between AI‐based and CRL‐based estimates without assuming that CRL‐based estimates represent a definitive true GA. In contrast, MAE does not distinguish between sources of error. Measurement error models address this limitation by separating operator effects, calibration differences and AI‐specific variation, thereby clarifying the structure of error and identifying key contributors. In our analysis, within‐operator variation appears to be a dominant source of error in AI‐based measurements, suggesting an important target for improving accuracy.

The main limitation of our study is the single‐center design. The scans were performed by a team of certified clinicians with extensive training and experience in first‐trimester ultrasound and measurement of CRL. This may have introduced a bias favoring CRL‐based pregnancy dating. Our study focused exclusively on first‐trimester dating, in which CRL is both the most accurate method and the standard of care in developed countries. The accuracy of biometry‐based pregnancy dating declines with advancing gestation, increasing the potential value of AI in the second and third trimesters, which was not addressed in our study. From a global health perspective, AI‐based pregnancy dating aims to broaden access to prenatal care in LMICs, in which first‐trimester ultrasound is often unavailable. However, our study was not designed to address the use of AI‐based GA estimation in LMICs or its use by novice ultrasound operators. Further validation studies are warranted to evaluate the accuracy and robustness of AI models in the second and third trimesters.

### Conclusions

Our data suggest high accuracy of AI‐based GA estimation in the first trimester, with a MAE of 2.0 days compared with CRL‐based dating. After adjusting for measurement error, the MAE of AI‐based estimation decreased to 1.6 days. When treating both CRL and AI symmetrically as the reference standard, the MAE was as low as 0.8 days for duplicate AI measurements. These numbers outperform literature benchmarks for the reproducibility of CRL.

Estimation of GA using AI holds considerable promise to reduce the dependency on skilled sonographers and the duration of ultrasound examinations in high‐resource settings, and to expand access to accurate pregnancy dating in low‐resource settings. However, determination of GA using CRL in the first trimester remains the gold standard in our study cohort, in which scans were performed by highly qualified sonographers.

Future studies should extend beyond the assessment of MAE and include analysis of the factors contributing to measurement errors. This may contribute to improving the development of deep‐learning models and improve AI‐based pregnancy dating.

## DISCLOSURE

The study was supported by a grant from the Fetal Medicine Foundation (UK Charity No.: 1037116). The AI system was provided free‐of‐charge by GE Healthcare. The funders had no involvement in the study design, in the collection, analysis and interpretation of data, in the writing of the report, or in the decision to submit the article for publication.

## Supporting information


**Appendix S1** Details of statistical analysis.
**Table S1** Mean absolute error in gestational age (days), according to maternal body mass index and ethnicity.

## Data Availability

The data that support the findings of this study are available from the corresponding author upon reasonable request.
