# Supplementary material for: Real‐time gestational‐age estimation at 11–13 weeks using artificial intelligence on non‐targeted ultrasound sweeps
Source: Ultrasound Obstet Gynecol. 2026 Sep 14;68(4):510–7. doi: 10.1002/uog.70335 (PMC13641732; doi:10.1002/uog.70335)
Supplement: Supplementary file 1 [file UOG-68-510-s001.docx]

**APPENDIX S1
Statistical Model**

To account for uncertainty in CRL-based gestational age estimates, a hierarchical measurement error model was fitted within a Bayesian framework, with the true gestational age (τ) treated as an unobserved latent variable.

Gestational age estimated from CRL was modeled as an imperfect measurement of τ, with hierarchical random effects included to account for both within- and between-sonographer variability. AI-based estimates were modeled analogously as functions of τ, with corresponding within- and between-operator random effects. A subject-specific random effect was included for AI measurements to capture additional measurement variability not explained by gestational age or operator effects.

Intercept and slope terms were included to allow for calibration differences between AI- and CRL-based estimates. For identifiability, the model was parameterised such that the scale of τ was anchored by CRL, with the mean CRL equal to τ. Weakly informative priors were used, with normal priors assigned to regression parameters and diffuse gamma priors assigned to precision (inverse variance) parameters.

Models were fitted in WinBUGS using Markov chain Monte Carlo methods with three chains of 200,000 iterations each, with the first 100,000 iterations discarded as burn-in. Convergence was assessed by inspection of trace plots and autocorrelations.

The WinBUGS model specification used for the analysis is available from the authors on request.

**Table S1.** Mean absolute error in gestational age (days) according to maternal BMI and ethnicity

|  |  | **MAE (days)** | **N (%)** | **p** |
| --- | --- | --- | --- | --- |
| BMI | <25 | 1.7 | 157 (55.1%) |  |
|  | 25-30 | 1.4 | 93 (32.6%) | 0.12 |
|  | >30 | 1.8 | 35 (12.3%) | 0.86 |
| Ethnicity | White | 1.6 | 218 76.5%) |  |
|  | Black | 1.7 | 31 (10.9%) | 0.90 |
|  | East Asian | 2.2 | 9 (3.2%) | 0.52 |
|  | Mixed | 1.5 | 8 (2.8%) | 0.87 |
|  | South Asian | 1.2 | 19 (6.7%) | 0.08 |

N (%) data represent number of participants per group. Data were analyzed using Kruskal-Wallis Test for independent samples and p<0.05 was considered statistically significant.
